# Supplementary material for: Splice-Junction-Based Mapping of Alternative Isoforms in the Human Proteome
Source: Cell Rep. Author manuscript; Available in PMC 2020 Jan 15. (PMC6961840; doi:10.1016/j.celrep.2019.11.026)
Supplement: 2 [file NIHMS1546469-supplement-2.zip › DF1/PXD010154/AdrenalGland/AdrenalGland_17_LRRC28_KPCSEASKPCGTVELELR.pdf]

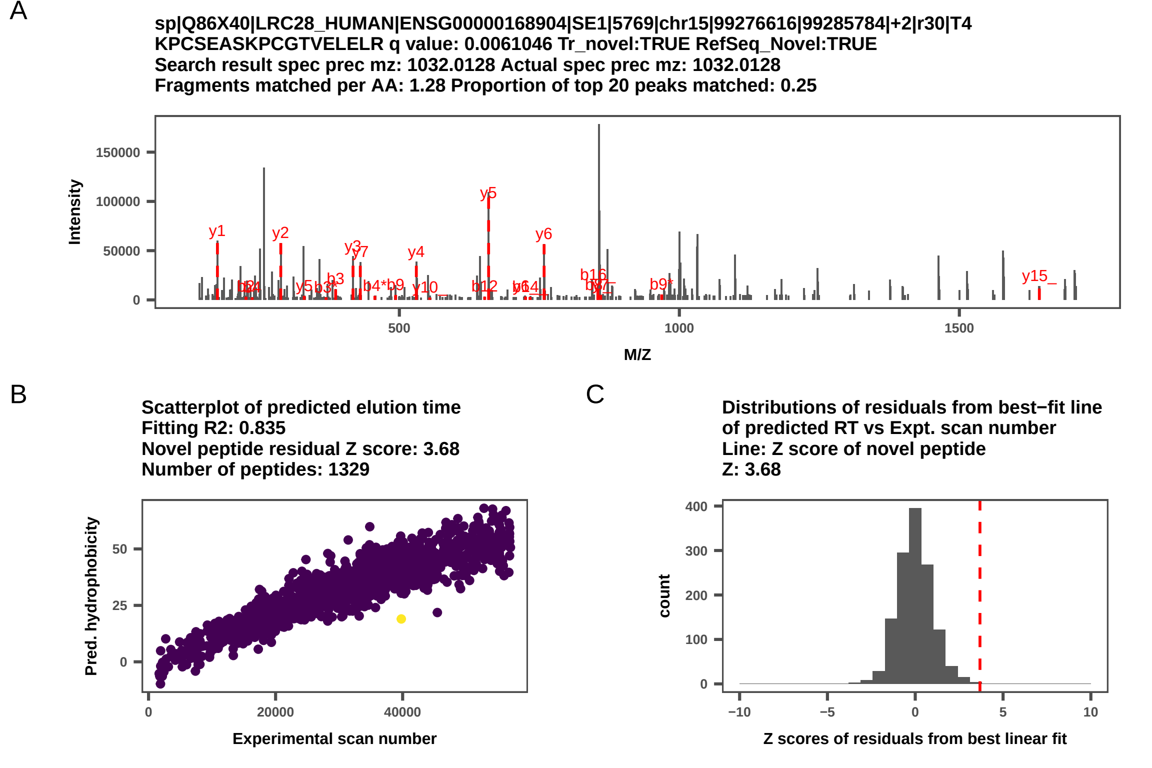
A
sp|Q86X40|LRC28_HUMAN|ENSG00000168904|SE1|5769|chr15|99276616|99285784|+2|r30|T4
KPCSEASKPCGTVELELR q value: 0.0061046 Tr_novel:TRUE RefSeq_Novel:TRUE
Search result spec prec mz: 1032.0128 Actual spec prec mz: 1032.0128
Fragments matched per AA: 1.28 Proportion of top 20 peaks matched: 0.25
Intensity
150000
100000
50000
0
y1
y2
y3
y7
y4
y5
y6
b3
b4*b9
y10_
b12
b16
b9*
y15_
500
1000
1500
M/Z
B
Scatterplot of predicted elution time
Fitting R2: 0.835
Novel peptide residual Z score: 3.68
Number of peptides: 1329
Pred. hydrophobicity
50
25
0
0
20000
40000
Experimental scan number
C
Distributions of residuals from best−fit line
of predicted RT vs Expt. scan number
Line: Z score of novel peptide
Z: 3.68
count
400
300
200
100
0
−10
−5
0
5
10
Z scores of residuals from best linear fit
